# Squid adjust their body color according to substrate

**DOI:** 10.1038/s41598-022-09209-6

**Published:** 2022-03-28

**Authors:** Ryuta Nakajima, Zdeněk Lajbner, Michael J. Kuba, Tamar Gutnick, Teresa L. Iglesias, Keishu Asada, Takahiro Nishibayashi, Jonathan Miller

**Affiliations:** 1 Physics and Biology Unit, Okinawa Institute of Science and Technology Graduate University (OIST), 1919-1 Tancha, Onna-son, Okinawa, 904-0945 Japan; 2 Department of Art and Design, University of Minnesota Duluth, 1201 Ordean Ct., Duluth, MN 55812 USA; 3 Animal Resources Section, Okinawa Institute of Science and Technology Graduate University (OIST), 1919-1 Tancha, Onna-son, Okinawa, 904-0945 Japan

**Keywords:** Ecology, Behavioural ecology

## Abstract

Coleoid cephalopods camouflage on timescales of seconds to match their visual surroundings. To date, studies of cephalopod camouflage-to-substrate have been focused primarily on benthic cuttlefish and octopus, because they are readily found sitting on the substrate. In contrast to benthic cephalopods, oval squid (*Sepioteuthis lessoniana* species complex) are semi-pelagic animals that spend most of their time in the water column. In this study, we demonstrate that in captivity, *S. lessoniana* Sp.2 (Shiro-ika, white-squid) from the Okinawa archipelago, Japan, adapts the coloration of their skin using their chromatophores according to the background substrate. We show that if the animal moves between substrates of different reflectivity, the body patterning is changed to match. Chromatophore matching to substrate has not been reported in any loliginid cephalopod under laboratory conditions. Adaptation of the chromatophore system to the bottom substrate in the laboratory is a novel experimental finding that establishes oval squid as laboratory model animals for further research on camouflage.

## Introduction

Camouflage is an important defense mechanism for many animals, both terrestrial and aquatic^1–4^. While for most animals, body patterns are fixed, and are some partially variable^5^, coleoid cephalopods such as octopuses, cuttlefish, and squid, display rapid dynamic pattern and texture changes^6–8^. This alteration in color, texture and associated posture and movement, in response to their visual surroundings can occur in less than a second^9–12^. To date, the study of cephalopod camouflage has focused chiefly on benthic species of cuttlefish and octopus^13^. Research on cuttlefish, both in the lab and in the wild, has described a wide variety of pattern and texture displays, among which are background matching, disruptive patterning, masquerading, countershading, and mimicry^9,14–19^. In contrast, because of the technical challenges of working with and filming octopus, most camouflage and background matching research in octopus has been conducted in the wild^20,21^.

The rapidity of cephalopod camouflage, when compared to other camouflaging animals, is due to the unique neural control of the color and texture changes. Color and pattern changes are achieved by neurally-controlled chromatophores, iridophores, and leucophores which are differentially distributed throughout their bodies^12,22^. Certain regions of the squid’s body have higher densities of leucophores, which may be obscured or exposed by the chromatophores^23^. The combined chromatic expression of these chromatophores, iridophores, and leucophores allows cephalopods to display a wide repertoire of body patterns, often specifically matched to camouflage them in visually diverse environments such as coral reefs^12,24,25^.

By altering their color, texture, body shape, motion patterns, cephalopods are capable of several types of camouflage strategies. Two major types of camouflage behavior that have been frequently observed in cephalopods are: (1) “camouflage in motion,” where the camouflage changes dynamically as the animal moves through the environment^26,27^; and (2) “situational motionless camouflage,” where the animal actively selects a site and camouflages at that site without moving from it. In general, motionless camouflage is more common in nature^5,28,29^ because visually enabled organisms are skilled at movement detection, and rapid changes in color and shape are impossible for most of animals^30–32^.

Camouflage in cuttlefish and octopuses has often been described in the literature, but squid camouflage is less studied^13^. Scientific studies of camouflage behavior in squid species are limited, as most species of squid are primarily pelagic, making both direct observation and laboratory studies challenging. To date, only the Caribbean reef squid, *Sepioteuthis sepioidea,* a semi-pelagic species, has been reported to exhibit background matching by mottled pattern, countershading, and translucency; disruptive coloration against soft coral; masquerading to soft coral; and mimicry to coral, in the wild^23,33^ and the longfin inshore squid, *Doryteuthis pealeii*, using disruptive pattern^34^. In addition to basic countershading reflex^35^, mesopelagic deep-sea squid, *Onychoteuthis banksii*, was reported to control pigmentation and transparency based on ambient light conditions to yield optimal countershading^36^ while bioluminescent squids are controlling countershading using photophores^37^.

Oval squid, *Sepioteuthis lessoniana* Férussac in Lesson, 1830, form a not fully defined species complex^38^. Three members of the species complex are traditionally and routinely recognized by fishermen in Okinawa, Japan, which are morphologically, molecularly, and behaviorally distinct^39–45^ awaiting a taxonomical revision^46^. Here, we have focused on *S. lessoniana* sp.2, also called “Shiro-ika” or white-squid. We use the name white-squid within this article in order to distinguish it clearly from other members of the *S. lessoniana* species complex that might be behaviorally distinct. Unlike any other squid of the Loliginidae family, white-squid are bred in captivity over multiple generations^47–49^ and thus represent an attractive model species for studying the distinctive biology of loliginid squids.

**Figure 1:**
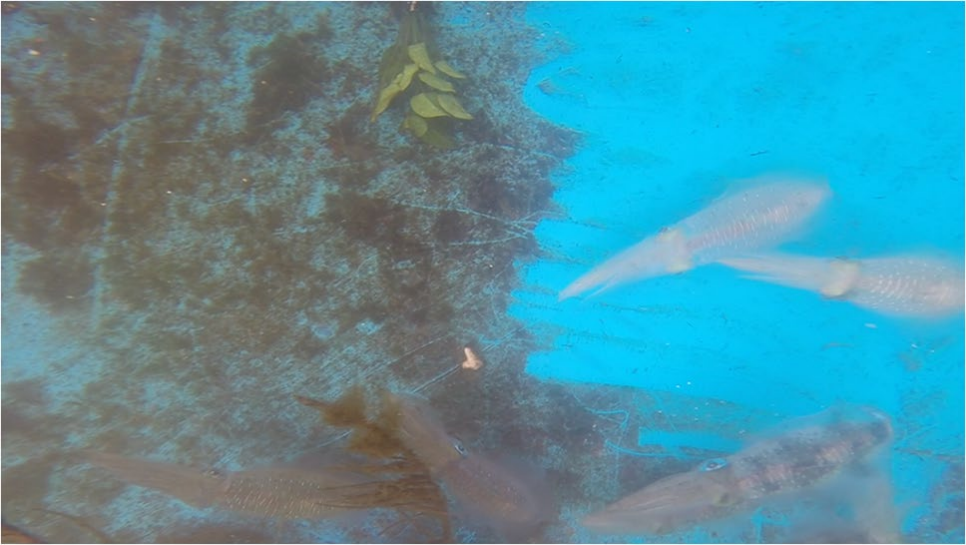
Squid substrate matching. The captive white-squid are swimming above a partially cleaned bottom of their tank, which is brighter than the uncleaned area, with dark and light body patterns over the matching dark and light substrate. This is a single frame from the Supplementary Video S1 of our initial observation in 2017.

In initial early observations of our laboratory housed white-squid, we recorded substrate color matching and camouflage in motion of adult white-squid, reared in captivity from wild collected eggs (Supplementary Video S1, Fig. 1; recorded in 2017). In the same year, a video of a squid from the *S. lessoniana* species complex was uploaded in the Seaunseen YouTube channel (https://www.youtube.com/watch?v=DzqhiRv-zOk; filmed by Renee Blundon in Kankadya Reef in Dar es Salaam, Tanzania). As the squid fed on passing fish it, at times, appeared to match the nearby substrate. These separate observations led us to design an experiment examining squid camouflage to substrate in motion in controlled laboratory conditions.

## Materials and methods

### Animal maintenance

This project was conducted with the approval of the Okinawa Institute of Science and Technology Graduate University Animal Welfare Committee (OIST; AAAE 2016-137). Second-generation laboratory-bred adult white-squid (*S. lessoniana* Sp.2, Shiro-ika) were used in this study.

Egg strings of white-squid were collected in August 2017 from intertidal seagrass bed in Tancha village on the west coast of Okinawa Island, Ryukyu Archipelago, Japan. The eggs were transported to the Okinawa Institute of Science and Technology Graduate University Marine Science Station (OMSS), where the white-squid life cycle was closed in a flow-through system^49^, and the white-squid were reared through multiple generations. Outdoor tanks were illuminated solely by natural light and fed by ocean water pumped through a plumbing system with a sand filter. Because of this minimal processing, the tank water was representative of the seawater conditions in the surrounding ocean that are recorded by the Japan Meteorological Agency (https://www.jma.go.jp/).

White-squid were fed up to four times a day, with food type depending on their age. Subadult and adult white-squid were offered defrosted subadult and adult silver-stripe round herring *Spratelloides gracilis* and occasionally live tiger prawn *Marsupenaeus japonicus*, mysid, larval anchovy, or ghost shrimp to enhance the animals’ feeding and hunting motivation, rather than as a daily source of nutrition. Dead white-squid, waste, and any remaining food particles or live food organisms were removed from the tank after feeding session or upon discovery. During maintenance, we observed that the white-squid color regularly matched substrate, starting from the initial generation that hatched in captivity from wild collected eggs, as shown on the video from November 2017, containing 8 white-squids in the tank (Supplementary Video S1).

### Data acquisition

We housed 3 sub-adult white-squids from the second laboratory-bred generation (two females and one male, hatched on August 5–8, 2018) starting from October 30, 2018 in a square-bottomed blue fiberglass tank with rounded corners and 156 cm lateral dimension (Aqua Culture system, Earth Corporation, Japan) sited outdoors. The depth of water was maintained at 40 cm. A SONY HD video camera (PXW-X70, SONY Corporation, Japan) was positioned above the water surface to record white-squid behavior and set at HD (1920 × 1080 at 30fps) resolution. We also placed GoPro (HERO6, GoPro inc. USA) cameras underwater for close-up HD videos (1980 × 1080 at 30fps) either perpendicular to the tank bottom on an outflow water pipe located at the center of the tank, or oriented parallel to the bottom surface at the bottom of the tank. The filming area was shaded by fabric to minimize light reflection from the water surface.

**Figure 2:**
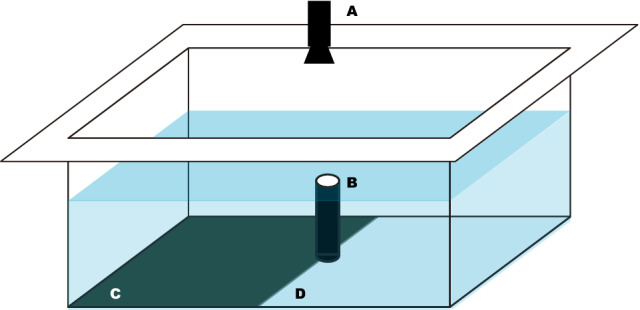
Diagram of the experimental arena. Video camera **(A)** is placed above the tank. Outflow tube **(B)** appears at the center of the tank. Algal cover creates the dark substrate in the tank **(C)**. The area where the bottom was cleaned, exposing the bright light blue color of the tank, which creates the light substrate **(D)**.

At the beginning of December, we removed algal growth from 50% of the tank bottom surface, yielding a distinctly reflective substrate on each 50% of the tank bottom: one with the light blue color of the fiberglass tank, and the other with the dark green color of algal growth (see Fig. 2). The experiment took place under overcast skies over on four following days at the end of December (Supplementary Table 1). Individuals are easy to recognize by size differences (Supplementary Tables 1, 2). For every trial of filming, a small amount of food (*Engraulis japonicus*) was fed to the white-squid from time-to-time to evaluate their health condition and confirm that it remained good throughout the experiment. During the experiment, white-squid were encouraged to cross the border between substrates by food or other attention-stimulating activities of the experimenter, initially on the first day also by a gentle submersion and a slow movement of PVC pipe (daily used for the tank cleaning) with an attached black ball on the opposite side of the tank than was the intended white-squid destination (Supplementary Video S2). This was no longer necessary by the second day of experiment when the white-squid reacted to mere presence and gesticulation of the experimenter. Over the 4 days, we were able to record 5 videos from above the tank and simultaneously side-view videos underwater (Supplementary Video S3, Supplementary Table 1).

### Data analysis

We examined three parameters: (1) white-squid coloration changing according to substrate, (2) duration of white-squid color-changing event, and (3) effect of swimming orientation on the position of white-squid in the environment during color-changing event.

The recordings were assessed at times after the white-squid had sufficiently recovered from initial disturbances generated by camera installation and other activities and started regularly crossing the divider between substrates under SONY camera.

**Figure 3:**
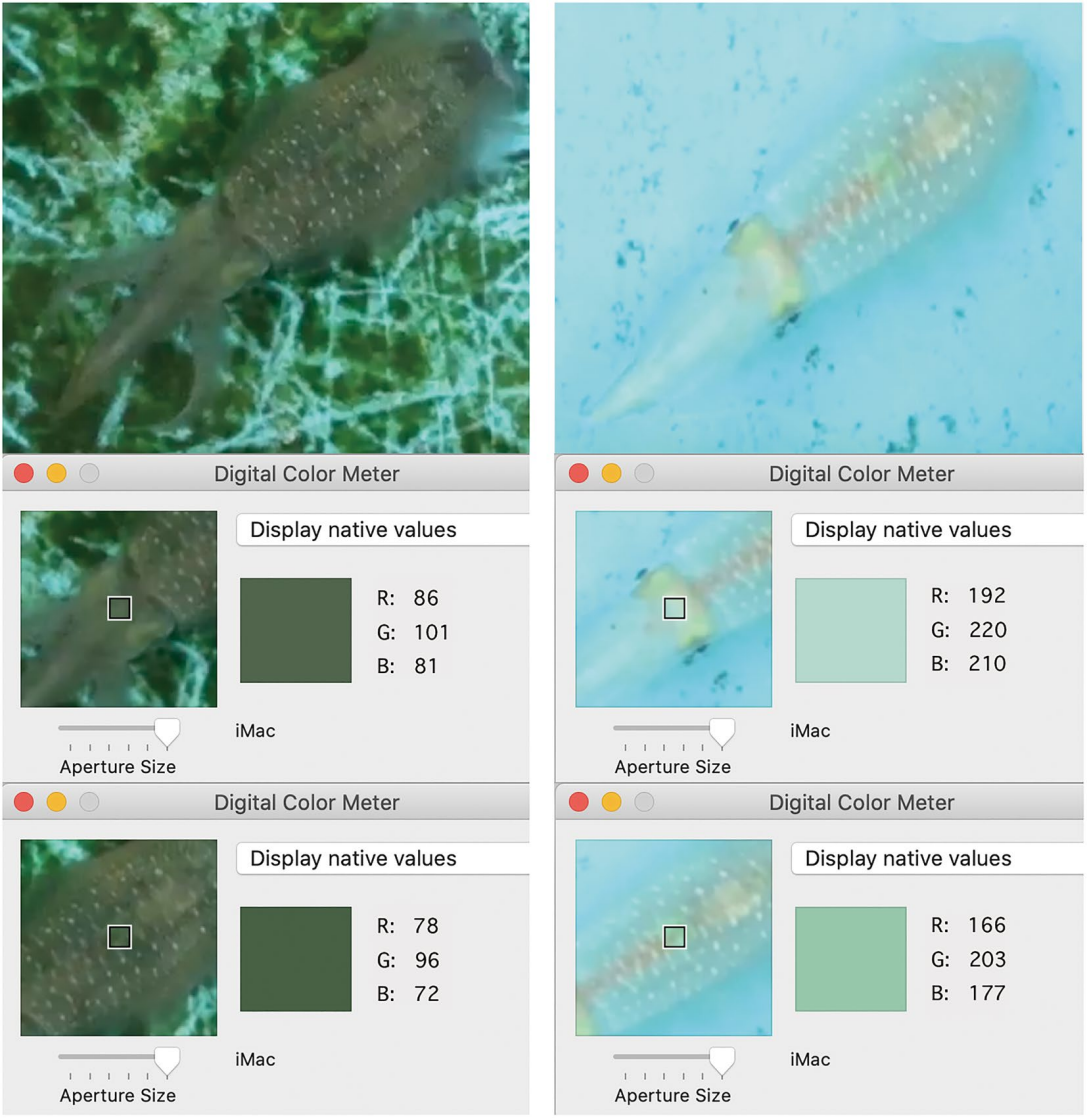
Example pictures of the subjects over dark or light substrates showing the RGB score as provided by the Digital Color Meter on iMac. When white-squid moved over different substrates, we measured the area between eyes of the animal, and the area right above the ink sac on the top view SONY camera recording (Supplementary Video S3), because these areas are never transparent and thus their color-change corresponds with activity of animal chromatophores.
  We examined correlations between RGB scores and the two measured areas by Pearson correlation coefficient (Supplementary Table 1). To confirm our general observation of color-change correlation with substrate, we applied a linear mixed-effect model (lmer) in the lme4 v. 1.1-21 package^50^ of R version 3.6.2^51^ in RStudio v. 1.2.5033^52^ with restricted maximum likelihood estimation of variance components, and type III Wald F-tests with Kenward-Roger degrees of freedom appropriate for finite sample size^53^. We modelled the substrate as a fixed effect, day and individual as random effects. White-squid bodies are semi-transparent; top-view SONY videos do not reach single chromatophore resolution, but side-view GoPro videos allowed us to distinguish the chromatophores and observe directly their expansions and contractions during experiment (Supplementary Video S2).
  The top-view SONY videos were further used to estimate the (2) duration of white-squid color-changing events, and the (3) effect of swimming orientation on the position of white-squid in the environment during color-changing event, because we noticed that the white-squid color does not always change at the same distance from the border between substrates. For purpose of these analyses, SONY recordings of 180 boundary crossing events were uploaded to Adobe Premier Pro 2020 for frame-by-frame analysis.
  In order to assess squid color-change, RGB scores of the ~ 1 cm^2^ region of skin located between the eyes of each white-squid were recorded (Fig. 3). Single frames were exported as jpeg image files. The images were examined using image analysis software, Image J^54^. All video frames remain in their original HD video format (1080 × 1920 pixel), which allowed constant and accurate plotting. The central axis of the white-squid crossing the line connecting the center of the eyes was plotted for all targeted white-squid on the images in pixel. The plotted location in pixels was converted to millimeters (mm) with Image J^54^. The 60 mm GoPro camera width was used to calculate pixel per mm in each video-record.

1. RGB scores of a ~ 1 cm^2^ (average 17.3 × 17.3, s.d. 2.11, pixel area) region of skin located between eyes and an equivalent region above the ink sac of each white-squid were measured by Digital Color Meter on iMac. The average RGB scores were calculated for each selected area independently (Fig. 3). These locations were selected because they are well defined, not transparent, and fully visible on the top view SONY camera recording. Each score was obtained within one second after a white-squid fully crossed the border between the two distinct substrates, as recorded by the cameras and displayed in SONY videos (Supplementary Table 1). The total lengths of the videos varied; to standardize we scored 10 min from each SONY video.

(2) For estimates of duration of squid color-changing events, we studied 10 events per animal per day of experiment (120 events in total). These events correspond with the 10 initial events per animal per day from the analysis of white-squid coloration changing according to the substrate (Supplementary Table 2). The beginning of the white-squid color-changing event was determined by the first video frame wherein color change is detected after a color remains stable for at least 15 video frames. The last frame was determined when the color change is completed, so that subsequently the white-squid color remains stable for at least 15 video frames. The number of frames from beginning to completion was counted and divided by 30 frames per second to determine each event's duration in seconds. We compared the duration of these color-changing events between days and animals by two-way ANOVA in R version 3.6.2^51^ in RStudio v. 1.2.5033^52^.

(3) Because of differences between days in swimming orientation, the effect of swimming orientation (Arms-leading or Mantle-leading) on the position of the white-squid in the environment during color-changing event was examined by focusing on 30 initial events per animal in the fourth day of experiment which provides a series of well-balanced events across both types of swimming orientation and both types of substrates. We estimated the effect of swimming orientation on the distance of the color-measured point on the white-squid, once its color-change is complete, from the nearest point of the border between substrates (see Supplementary Table 2) by a linear mixed-effect model (lmer) in the lme4 v. 1.1-21 package^50^ of R version 3.6.2^51^ in RStudio v. 1.2.5033^52^ with restricted maximum likelihood estimation of variance components, and type III Wald F-tests with Kenward-Roger degrees of freedom appropriate for finite sample size^53^. We modelled the swimming orientation and type of substrate as fixed effects, individual as a random effect.

### Ethics approval

All applicable international, national, and/or institutional guidelines for animal testing, animal care and use of animals were followed by the authors.

## Results

### White-squid coloration changing according to substrate

**Figure 4:**
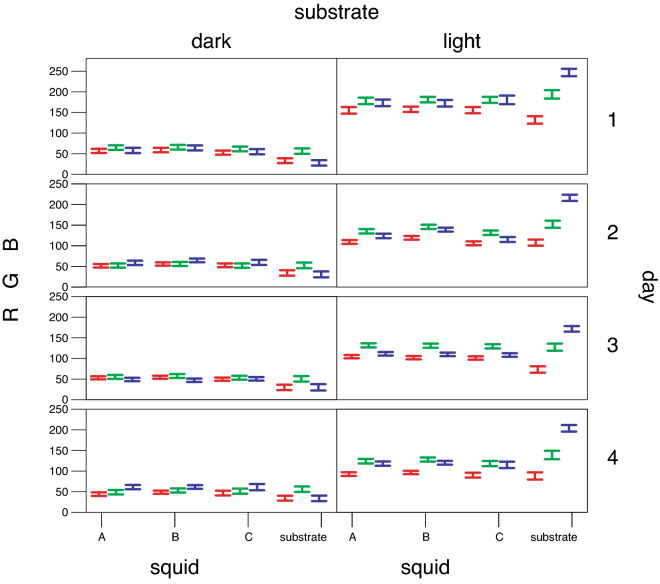
Average head RGB values per day (1–4) per white-squid (**A–C**) and the substrate on dark and light substrate areas. Error-bars represent a 95% confidence interval for the mean.

Our selected mathematical model revealed a highly significant interaction between the fixed effect of substrate (Fig. 3) and all the measured RGB color channel values (p < 0.0001) on the head that appear all highly positively correlated with each other and with the mantle (r > 0.94, p < 0.0001; Supplementary Table 1) and matching the destination substrate (Fig. 4).

### Duration of white-squid color-changing event

White-squid changed their color during all examined boundary-crossing events, and all examined color-changing events are corresponding to these boundary-crossing events. The shortest measured duration of a white-squid color-changing event was approximately a third of a second, but two seconds was the average duration of white-squid color-changing event (Supplementary Table 2). We found significant differences in the duration of body pattern transformation between days covered by our experiments (d.f. = 3, F = 4.831, p = 0.0034), but we did not detect significant differences between individuals in our experiment over all days. The average velocity of white-squid during the color-changing event was approximately 0.16 m/s.

### Effect of swimming orientation on the position of white-squid in the environment during color-changing event

We found that Mantle-leading swimming orientation resulted in the white-squid color-change occurring at a significantly (p < 0.0001) shorter distance from the substrate divider (mean = 13 ± 8 cm) when compared to Arm-leading swimming orientation (mean = 25 ± 8 cm), but there was no difference between traveling from light to dark or from dark to light substrate (Table 1, Supplementary Table 2).

**Table 1:** Mixed model examining the effect of swimming orientation and substrate type on the white-squid location at the end of color-changing event, as a response variable.

	*F*	d.f	P*(*> *F)*
Orientation	42.7746	1	4.349e−09***
Substrate	2.1507	1	0.1461835
Orientation:substrate	1.9760	1	0.1634171

Swimming orientation type (arm-leading, mantle-leading) and substrate type (light, dark) were modelled as fixed effects. Inter-individual variation was modelled as a random effect. Asterisks indicate statistical significance.

## Discussion

Although camouflage in squids has long been thought to be based on transparency, we demonstrated that they are also using chromatophores to adjust their color according to substrate. The animals in our experiment consistently (p < 0.0001) change their coloration while crossing between two distinct substrates. Although squid might have separate neural pathways to control their chromatic patterns on different body parts^37,55,56^, we observed a highly significant positive correlation between coloration on the head and mantle of camouflaging squid (r > 0.94, p < 0.0001; Supplementary Table 1), similarly to Josef et al. ’s^26,27^ observations in cuttlefish.

In a pelagic environment, the distinction between camouflage-to-substrate in motion behavior and countershading, which is widely associated with squids in the literature^35,57^, may be semantic. White-squid, however, reproduce in the optically heterogenous habitat of coral lagoons and seagrass meadows, returning to very shallow waters at times of maturity. In the latter habitat, countershading would be less effective than the camouflage-to-substrate capability of white-squid observed.

Squids face many different predators during their ontogeny and their defensive strategies change accordingly^58^. Our study focuses on mature or nearly mature animals. It has yet to be established when and how camouflage-to-substrate behavior emerges and/or changes during squid ontogenesis. The physiological factors that might be responsible for this behavior, and how environmental factors affect it have yet to be clearly defined.

The position of white-squid in the environment during color-changing events is significantly affected by squid swimming orientation (p < 0.0001; Table 1, Supplementary Table 2). The shortest white-squid color-changing event duration recorded by us in this study (0.3 s) corresponds with published single chromatophore expansion/contraction durations in loliginid squids^59–61^, but on average, the color-changing events in our study took four times longer. This variable and relatively long duration for the color-changing process which begins and is completed often centimeters from the divider (Supplementary Table 2) suggests that although squids anticipate the upcoming background, evidenced by becoming paler or darker before entering the light or dark substrate respectively, the final color is usually fully determined once the divider has been crossed, similarly to cuttlefish^26,27^. Alternatively, in our experiment, the duration of color-changing events might be affected by various external factors that have not been fully controlled, such as the mildness of stress induced by presence of experimenter, time since feeding, weather, natural lighting, water temperature, turbidity, chemistry, etc. Based on subjective visual examination of the video-recordings, we hypothesize that social interactions also influence squid camouflage behavior, including duration of body pattern transformation. Nonetheless, squids in our experimental setup are clearly changing color while in motion according to their proximity to the different substrates, regardless of coloration of their congeners farther away in the tank.

White-squid possess a rare combination of semitransparency and the ability to change body color via chromatophores (metachrosis) which enables them to successfully inhabit both pelagic and reef environments. Therefore, it represents an attractive model organism for studying the ecology, evolution, and neurobiology of versatile, dynamic camouflage.

## Supplementary Information

Supplementary Table 1.

Supplementary Table 2.

Supplementary Video S1.

Supplementary Video S2.

Supplementary Video S3.

## Data Availability

All data generated and analysed during this study are included in this published article [and its supplementary information files].

## Code availability

No custom code was used in this study.
